# Identification of a region required for TSC1 stability by functional analysis of *TSC1 *missense mutations found in individuals with tuberous sclerosis complex

**DOI:** 10.1186/1471-2350-10-88

**Published:** 2009-09-11

**Authors:** Melika Mozaffari, Marianne Hoogeveen-Westerveld, David Kwiatkowski, Julian Sampson, Rosemary Ekong, Sue Povey, Johan T den Dunnen, Ans van den Ouweland, Dicky Halley, Mark Nellist

**Affiliations:** 1 Department of Clinical Genetics, Erasmus Medical Centre, 3015 GE Rotterdam, The Netherlands; 2 Division of Experimental Medicine and Medical Oncology, Brigham and Womens Hospital, Boston MA 02115, USA; 3 Institute of Medical Genetics, University of Wales College of Medicine, Heath Park, Cardiff CF4 4XN, UK; 4 Research Department of Genetics, Evolution and Environment, University College London Wolfson House, 4 Stephenson Way, London, NW1 2HE, UK; 5 Department of Human and Clinical Genetics, Leiden University Medical Centre, 2333ZC Leiden, The Netherlands

## Abstract

**Background:**

Tuberous sclerosis complex (TSC) is an autosomal dominant disorder characterised by the development of hamartomas in a variety of organs and tissues. The disease is caused by mutations in either the *TSC1 *gene on chromosome 9q34, or the *TSC2 *gene on chromosome 16p13.3. The *TSC1 *and *TSC2 *gene products, TSC1 and TSC2, form a protein complex that inhibits signal transduction to the downstream effectors of the mammalian target of rapamycin (mTOR). Recently it has been shown that missense mutations to the *TSC1 *gene can cause TSC.

**Methods:**

We have used *in vitro *biochemical assays to investigate the effects on TSC1 function of *TSC1 *missense variants submitted to the Leiden Open Variation Database.

**Results:**

We identified specific substitutions between amino acids 50 and 190 in the N-terminal region of TSC1 that result in reduced steady state levels of the protein and lead to increased mTOR signalling.

**Conclusion:**

Our results suggest that amino acid residues within the N-terminal region of TSC1 are important for TSC1 function and for maintaining the activity of the TSC1-TSC2 complex.

## Background

Tuberous sclerosis complex (TSC) is an autosomal dominant disorder characterised by the development of hamartomas in a variety of organs and tissues, including the brain, skin and kidneys [1,2]. Mutations in either the *TSC1 *gene on chromosome 9q34 [3], or the *TSC2 *gene on chromosome 16p13.3 [4] cause TSC. In most studies, 75 - 85% of individuals with TSC have been shown to carry a germ-line *TSC1 *or *TSC2 *mutation [5-9] and a further 5 - 10% carry *TSC1 *or *TSC2 *variants where it is not absolutely clear from the genetic data whether the change is disease-causing (a pathogenic variant), or not (a neutral variant). To determine whether these unclassified variants are disease-causing, the effect of the changes on protein function can be investigated [10,11].

The *TSC1 *and *TSC2 *gene products, TSC1 and TSC2, interact to form a protein complex [12]. TSC2 contains a GTPase activating protein (GAP) domain and acts on the rheb GTPase to inhibit rheb-GTP-dependent stimulation of the mammalian target of rapamycin (mTOR) [13]. The exact role of TSC1 in the TSC1-TSC2 complex is less clear, but it appears to be required for maintaining the stability, activity and correct intracellular localisation of the complex [14]. Inactivation of the TSC1-TSC2 complex results in activation of mTOR and phosphorylation of the mTOR targets p70 S6 kinase (S6K), ribosomal protein S6 and 4E-BP1 [15]. The effects of amino acid changes to TSC1 or TSC2 on TSC1-TSC2 complex formation, on the activation of rheb GTPase activity by the complex, and on the phosphorylation status of S6K and S6 can be determined [10,11,16]. Pathogenic missense changes towards the N-terminus of TSC2 prevent formation of the TSC1-TSC2 complex, while missense changes towards the C-terminus do not prevent TSC1-TSC2 binding but disrupt the rhebGAP activity of TSC2 directly [10]. Pathogenic TSC1 missense changes are rare [5-9]. However, recent studies of TSC1 missense variants identified in bladder cancers [17] and in patients with TSC [11] have shown that TSC1 amino acid substitutions can be pathogenic, reducing steady state levels of TSC1 and leading to increased mTOR activity.

Here, we test 13 *TSC1 *variants identified during mutation screening of individuals with TSC. Our analysis confirms that pathogenic *TSC1 *missense mutations reduce steady state levels of TSC1, and result in increased mTOR signalling. Furthermore, we find that the intracellular localisation of pathogenic and neutral TSC1 variants is distinct. The pathogenic TSC1 amino acid substitutions are clustered within the conserved, hydrophobic N-terminal region of TSC1, indicating that this region plays an important role in TSC1 function.

## Methods

### Comparative analysis of TSC1 amino acid substitutions

*TSC1 *missense variants identified in individuals with TSC, or suspected of having TSC, and submitted to the LOVD *TSC1 *mutation database [18,19] were chosen for analysis. To predict whether the variants were likely to be pathogenic, the amino acid substitutions were evaluated using the BLOSUM 62 and Grantham matrices [20,21], and a multiple sequence alignment analysis was performed using SIFT software [22,23]. Hydrophobicity plots and secondary structure predictions were generated using DNAMAN (Lynnon BioSoft), SABLE [24] and PSIPRED [25] software. To determine whether the identified changes were likely to have an effect on splicing, 3 different splice-site prediction programs were used [26-28].

### Generation of constructs and antisera

Expression constructs encoding myc-tagged TSC1 variants were derived using the QuikChange site-directed mutagenesis kit (Stratagene, La Jolla, CA, U.S.A.). In each case the complete open reading frame of the mutated construct was verified by sequence analysis. Other constructs used in this study have been described previously [10,11]. Antibodies were described previously [12] or purchased from Cell Signaling Technology (Danvers, MA, U.S.A.), except for a mouse monoclonal antibody against TSC2 which was purchased from Zymed Laboratories (San Francisco, CA, U.S.A.) and a rabbit polyclonal antibody against ubiquitin which was purchased from DAKO (Glostrup, Denmark).

### Effects of TSC1 variants on S6K T389 phosphorylation

Immunoblot analysis of S6K T389 phosphorylation in cells over-expressing TSC1 variants was performed as described previously [11]. Briefly, 80% confluent HEK 293T cells in 24-well cell culture dishes were transfected with a 4:2:1 mixture of the TSC1, TSC2 and S6K expression constructs (0.7 μg DNA total) in 50 μl Dulbecco's modified Eagle medium (DMEM) containing 2.1 μg polyethyleneimine (Polysciences, Warrington, PA, U.S.A.). Where necessary, an empty expression vector (pcDNA3; Invitrogen, Carlsbad, CA, U.S.A.) was added to make up the total amount of DNA. Twenty-four hours after transfection the cells were washed with cold PBS and lysed in 75 μl 50 mM Tris-HCl pH 8.0, 150 mM NaCl, 50 mM NaF and 1% Triton X100 containing protease inhibitors (Complete, Roche Molecular Biochemicals, Woerden, The Netherlands). After centrifugation (10 000 g, 10 minutes, 4°C), the supernatant and pellet fractions were recovered for immunoblot analysis. The pellet fractions were resuspended in 100 μl 62.5 mM Tris-HCl pH 6.8, 10% glycerol, 300 mM 2-mercaptoethanol, 2% SDS and sonicated (8 μm, 15 seconds, 4°C) prior to gel electrophoresis. Samples were run on 4-12% SDS-PAGE Criterion gradient gels (BioRad, Hercules, CA, U.S.A.) and blotted onto nitrocellulose. Blots were analysed using near infra-red fluorescent detection on an Odyssey™ Infrared Imager (169 μm resolution, medium quality with 0 mm focus offset) (Li-Cor Biosciences, Lincoln, NE, U.S.A.). The integrated intensities of the protein bands relative to the wild-type TSC1 values were determined in at least 3 separate experiments using the Odyssey™ software (default settings with background correction; 3 pixel width border average method). Signal intensities were compared as described previously [29].

### Proteosome-mediated degradation of TSC1 variants

HEK 293T cells were transfected with expression constructs encoding TSC2, S6K and the different TSC1 variants. Twenty-four hours after transfection the culture medium was replaced with DMEM containing either 42 μM MG-132 (Sigma-Aldrich, St. Louis, MO, U.S.A.) or vehicle control. After 4 hours, insulin (200 nM; Sigma-Aldrich) or vehicle control was added to the culture medium and, after a further 30 minute incubation, the cells were harvested and analysed by immunoblotting, as before.

### Immunofluorescent detection of TSC1 variants

HEK 293T cells were seeded onto glass coverslips coated with poly-L-lysine (Sigma-Aldrich), transfected with expression constructs encoding the TSC1 variants and processed for immunofluorescent microscopy as described previously [12]. Fixed, permeabilised cells were incubated with a rabbit polyclonal antibody specific for TSC2 [12] and a mouse monoclonal antibody specific for the TSC1 C-terminal myc epitope tag (9B11, Cell Signaling Technology), followed by fluorescein isothiocyanate- and Cy3-coupled secondary antibodies against mouse and rabbit immunoglobulins (DAKO) respectively. Cells were studied using a Leica DM RXA microscope and Image Pro-Plus version 6 image analysis software.

## Results

### Comparative analysis of TSC1 amino acid substitutions

We selected 13 TSC1 amino acid substitutions (*TSC1 *c.149T>C (p.L50P), c.153A>C (p.E51D), c.182T>C (p.L61P), c.278T>G (p.L93R), c.397G>T (p.V133F), c.568C>T (p.R190C), c.569G>C (p.R190P), c.1001C>T (p.S334L), c.1433A>G (p.E478G), c.1648C>G (p.Q550E), c.1974C>G (p.D658E), c.1976C>T (p.A659V) and c.2420T>C (p.I807T)) that were either possible pathogenic changes, or could not be excluded as neutral variants [18] (Table 1). In this group there was one confirmed *de novo *change, *TSC1 *c.182T>C (p.L61P), that was very likely to be pathogenic. In one individual with TSC, 2 *TSC1 *missense changes, c.149T>C (p.L50P) and c.2420T>C (p.I807T), were reported. To establish whether either of these changes was responsible for TSC, or whether the combination of both changes was required to inactivate TSC1, we tested a L50P-I807T double variant as well as the L50P and I807T single variants.

**Table 1:** Summary of the functional analysis of TSC1 missense variants from the LOVD *TSC1 *mutation database

**Nucleotide change**	**Exon**	**Amino acid substitution**	**SIFT prediction**	**Effect on TSC1 function?** **(t-test)**	**Conclusion**
c.149T>C	4	p.L50P	not tolerated	yes (p = 0.03)	pathogenic
c.153A>C	4	p.E51D	tolerated	no (p = 0.3)	neutral variant
c.182T>C	4	p.L61P	not tolerated	yes (p = 0.002)	pathogenic
c.278T>G	5	p.L93R	not tolerated	yes (p = 0.03)	pathogenic
c.397G>T	6	p.V133F	not tolerated	yes (p = 0.005)	pathogenic
c.569G>C	7	p.R190P	not tolerated	yes (p = 0.004)	pathogenic
c.568C>T	7	p.R190C	not tolerated	no (p = 0.19)	neutral variant
c.1001C>T	10	p.S334L	tolerated	no (p = 0.19)	neutral variant
c.1433A>G	14	p.E478G	tolerated	no (p = 0.6)	possible splice mutation
c.1648C>G	15	p.Q550E	tolerated	no (p = 0.9)	neutral variant
c.1974C>G	15	p.D658E	tolerated	no (p = 0.13)	neutral variant
c.1976C>T	15	p.A659V	tolerated	no (p = 0.1)	neutral variant
c.2420T>C	19	p.I807T	tolerated	no (p = 0.18)	neutral variant

**Figure 1:**
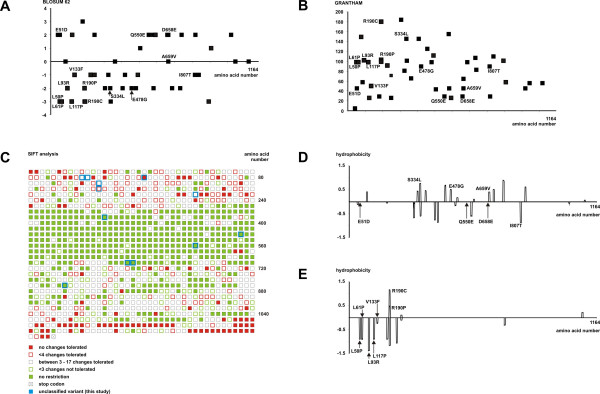
**Predicted effects of amino acid substitutions on TSC1**. (A) Plot of BLOSUM 62 scores for substitutions in the LOVD *TSC1 *mutation database. Positive values represent conservative substitutions, negative values represent non-conservative substitutions. Substitutions investigated in this study are indicated. (B) Plot of Grantham scores for substitutions in the LOVD *TSC1 *mutation database. The higher the Grantham score, the less conservative the substitution. Substitutions investigated in this study are indicated. (C) SIFT analysis of TSC1. Each amino acid is represented by a box. Solid green boxes represent positions that are completely tolerant (all substitutions possible); open green boxes represent positions where 1 or 2 substitutions are not tolerated. Solid red boxes represent intolerant positions (no substitutions tolerated); open red boxes represent positions where 3 or fewer substitutions are tolerated. Empty boxes represent positions where between 3 and 17 substitutions are tolerated. The positions of substitutions tested in this study are indicated in blue. (D) Comparative hydrophobicity profile of wild-type TSC1 with variants predicted by SIFT analysis to be tolerated. Hydrophobicity values were calculated for each amino acid using DNAMAN software (default parameters). Differences in the predicted hydrophobicity per amino acid were plotted. Values > 0 indicate an increase in hydrophobicity of the variant relative to wild-type; values < 0 indicate a decrease. Variants analysed as part of this study are indicated. (E). Comparative hydrophobicity profile of wild-type TSC1 with variants predicted by SIFT analysis not to be tolerated.

To predict whether the amino acid substitutions were likely to be pathogenic, we determined the scores of the BLOSUM 62 [20] and Grantham [21] amino acid substitution matrices for all the missense variants in the LOVD *TSC1 *database (Figure 1A and 1B). These scores give an indication of the differences between amino acid pairs based on amino acid composition, polarity and molecular volume, as well as substitution frequencies. Both matrices indicated that the amino acid substitutions listed in the LOVD *TSC1 *database in general become more conservative towards the C-terminal of TSC1. We also performed a SIFT analysis [23] using a multiple sequence alignment of TSC1 derived from 14 different species (human, chimpanzee, maccaca, cow, dog, horse, mouse, rat, chicken, pufferfish, honey bee, fruitfly, mosquito and fission yeast). The SIFT algorithm combines information from both the chemical structure of the individual amino acids and the evolutionary conservation of the protein to predict for each amino acid residue which substitutions can be tolerated by the protein. The results of the SIFT analysis are shown in Figure 1C and Table 1. Overall, the N-terminal (amino acids 1 - 270) and C-terminal (amino acids 680 - 1164) regions of TSC1 did not tolerate amino acid substitutions while the central region (amino acids 270 - 680) was predicted to be more tolerant (Figure 1C). Of the 13 substitutions selected for this study, 7 were predicted to be tolerated and therefore unlikely to be pathogenic (Table 1). Of these, 5 were located in the central, substitution-tolerant region of TSC1 (amino acids 270 - 680). Six substitutions were predicted not to be tolerated and therefore likely to be pathogenic. All of these changes were located in the substitution-intolerant N-terminal region (amino acids 1 - 270). Finally, we investigated the effect of the substitutions on the hydrophobicity of TSC1 (Figure 1D and 1E). Three substitutions, p.E51D, p.Q550E and p.D658E, had no significant effect. These were all predicted by the SIFT algorithm to be tolerated changes. Of the remaining tolerated changes, p.S334L, p.E478G and p.A659V increased the hydrophobicity, while the p.I807T substitution had the opposite effect (Figure 1D). In the group of changes not tolerated by the SIFT analysis, the p.L50P, p.L61P, p.L93R and p.V133F substitutions decreased the hydrophobicity while the p.R190C and p.R190P substitutions increased the hydrophobicity (Figure 1E).

Splice site prediction analysis of the *TSC1 *nucleotide changes was performed using 3 independent splice site prediction programs. Neither NetGene2 [26] nor Human Splicing Finder [28] predicted any splicing defects for any of the 13 changes. In contrast, NNSPLICE 0.9 [27] predicted that the *TSC1 *c.1433A>G (p.E478G) change would result in the creation of an alternative splice donor site 6 nucleotides downstream of the wild-type exon 14 donor site. Furthermore, the wild-type site was no longer recognised as a splice donor site by the NNSPLICE 0.9 algorithm. If this prediction is correct, the new splice donor site created by the *TSC1 *c.1433A>G change would result in the insertion of 2 extra codons (encoding amino acids GN) between codons 479 and 480, in addition to the p.E478G substitution.

### Functional analysis of TSC1 amino acid substitutions

**Figure 2:**
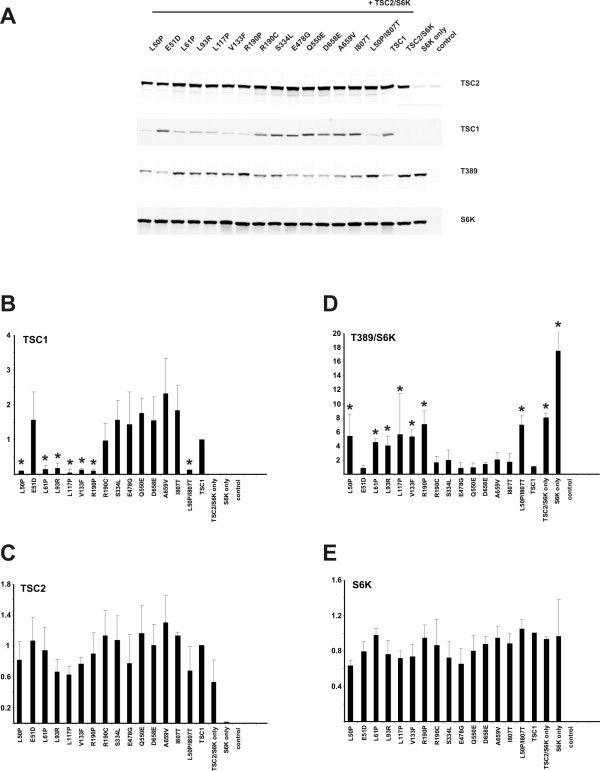
**Immunoblot analysis of TSC1 variants**. (A) Cells expressing the TSC1 variants and control cells expressing wild-type TSC1 and S6K only (TSC1/S6K), TSC2 and S6K only (TSC2/S6K), S6K only (S6K) or empty vector only (control) were analysed by immunoblotting. (B) Quantification of the immunoblot signals for the TSC1 variants. The integrated intensities of the signals for each variant, relative to wild-type TSC1, were determined in 3 independent experiments. Standard deviations are indicated. Signals significantly reduced compared to wild-type (unpaired t-test, p values < 0.05) are indicated with asterisks. (C) Quantification of the TSC2 signals. The TSC2 signals in the presence of the different TSC1 variants were not significantly different from the signal in the presence of wild-type TSC1 (unpaired t-test, p values >0.05). (D) Inhibition of S6K T389 phosphorylation in the presence of the TSC1 variants. The ratio of the S6K T389 phosphorylation signal to the total S6K signal (T389/S6K) was determined relative to wild-type TSC1 (wild-type TSC1 T389/S6K ratio = 1). Standard deviations are indicated. Variants with a significantly increased T389/S6K ratio (unpaired t-test p values < 0.05) are indicated with asterisks. (E) Quantification of the total S6K signals. The total S6K signals in the presence of the different variants were not significantly different to wild-type TSC1 (unpaired t-test, p values >0.05).

We characterised the effects of the 13 TSC1 single missense variants and the L50P/I807T double variant on the activity of the TSC1-TSC2 complex. We compared the 14 variants to wild-type TSC1 and the TSC1-L117P pathogenic variant [11]. As shown in Figure 2, the L50P, L61P, L93R, V133F and R190P amino acid substitutions all resulted in reduced levels of TSC1 (Figure 2A and 2B) and increased mTOR activity, as estimated by the T389 phosphorylation status of S6K (Figure 2A and 2D). These substitutions all had the same effect on TSC1 as the previously-characterised L117P substitution [11]. In each case, the T389/S6K ratio was significantly different from wild-type TSC1 (unpaired t-test p values all < 0.05; Table 1). We did not observe any differences between the L50P variant and the L50P/I807T double variant. Both variants were detected at low levels and did not inhibit S6K phosphorylation as effectively as wild-type TSC1.

The E51D, R190C, S334L, E478G, Q550E, D658E, A659V and I807T variants were detected at comparable levels to wild-type TSC1 (Figure 2A and 2B) and S6K T389 phosphorylation was reduced to the same levels as in the presence of wild-type TSC1 (Figure 2A and 2D). In each case, there was no significance difference in the T389/S6K ratio compared to wild-type TSC1 (unpaired t-test p values > 0.05; Table 1).

### TSC1 variants are degraded by the proteosome

We considered two possible reasons for why the TSC1 L50P, L61P, L93R, V133F, R190P and L50P/I807T variants were detected at low levels. One possibility was that the variants were insoluble and therefore not present in the cell lysate fraction analysed by immunoblotting. We analysed the insoluble post cell lysis pellet fractions, but did not detect any of the TSC1 variants in these fractions (data not shown).

**Figure 3:**
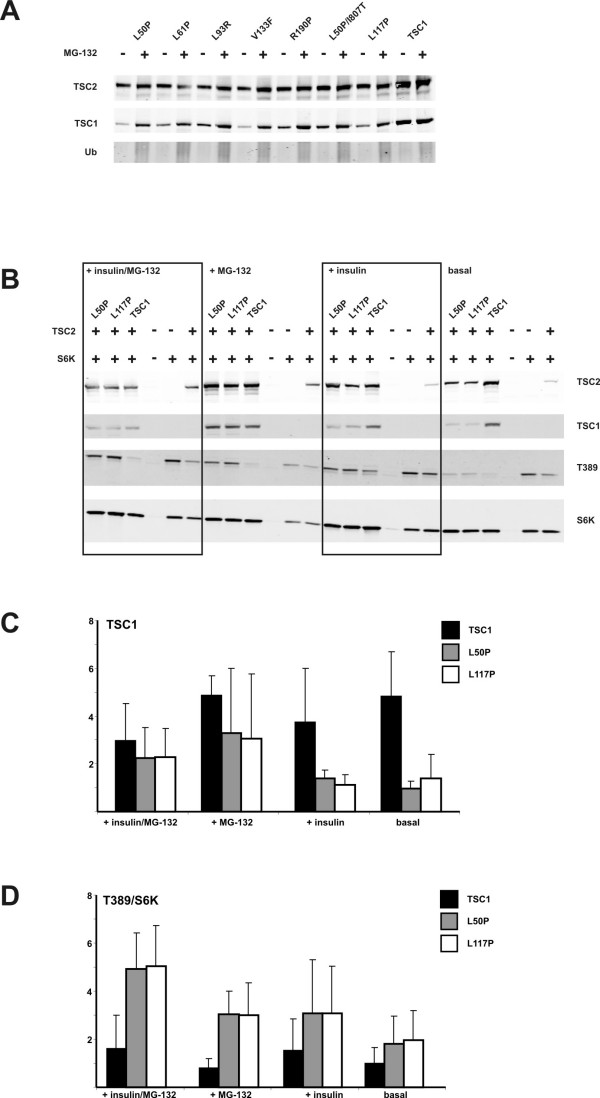
**Proteosome-mediated degradation of pathogenic TSC1 variants**. (A) Immunoblot showing levels of TSC2, wild-type TSC1 and pathogenic TSC1 variants in cells treated with 42 μM MG-132 for 4 hours, or left untreated. The blot was incubated with an antibody against ubiquitin (Ub) to show the effect of the MG-132 treatment. (B) Immunoblot showing levels of TSC2, wild-type TSC1 and the L50P and L117P variants, S6K and T389-phosphorylated S6K (T389) in cells treated with 42 μM MG-132 and 200 nM insulin (+ insulin/MG-132), 42 μM MG-132 only (+ MG-132), 200 nM insulin only (+ insulin), or left untreated (basal). (C) Quantification of the immunoblot signals, showing MG-132-dependent inhibition of proteosome-mediated degradation of the TSC1 L50P and L117P variants. The mean integrated intensities (arbitrary units) of the signals for wild-type TSC1 (black), and the L50P (grey) and L117P (white) variants are shown. MG-132 treatment resulted in a relative increase in the signals for the L50P and L117P variants compared to wild-type TSC1 in both insulin stimulated and unstimulated cells. (D) Quantification of the immunoblot signals showing increased S6K T389 phosphorylation in MG-132 treated cells expressing the L50P or L117P variants. S6K T389 phosphorylation was determined as in Figure 2. (T389/S6K ratio). Compared to MG-132-treated cells expressing wild-type TSC1, MG-132 treatment of cells expressing the L50P and L117P variants increased the T389/S6K ratio.

The second possibility was that the L50P, L61P, L93R, V133F, R190P and L50P/I807T variants were subject to rapid turn-over and degradation. Therefore, we treated cells expressing wild-type TSC1 or the L50P, L61P, L93R, V133F, R190P, L50P/I807T or L117P variants with the proteosome-inhibitor MG-132 [30]. After 4 hours treatment we observed an increase in the L50P, L61P, L93R, V133F, R190P, L50P/I807T and L117P signals when cells were treated with MG-132 (Figure 3A).

We estimated mTOR activity in MG-132-treated cells by measuring the T389 phosphorylation status of S6K in cells expressing the L50P or L117P variants and treated with MG-132 and/or insulin (Figure 3B-3D). The L50P and L117P signals were increased relative to the TSC1 wild-type signals in unstimulated cells treated with MG-132 as well as in cells treated with MG-132 and stimulated with 200 nM insulin for 30 minutes (Figure 3B and 3C). In (unstimulated and insulin stimulated) cells not treated with MG-132, wild-type TSC1 was detected at higher levels than the L50P and L117P variants, consistent with the analysis shown in Figure 2. In untreated cells, S6K T389 phosphorylation was increased in cells expressing the L50P and L117P variants, compared to cells expressing wild-type TSC1, consistent with the analysis shown in Figure 2. MG-132 treatment did not affect the detected levels of total S6K (Figure 3A), but in cells expressing the L50P and L117P variants, S6K T389 phosphorylation was increased compared to cells expressing wild-type TSC1, in both insulin-stimulated and unstimulated cells (Figure 3B and 3D).

### Intracellular localisation of the TSC1 variants

**Figure 4:**
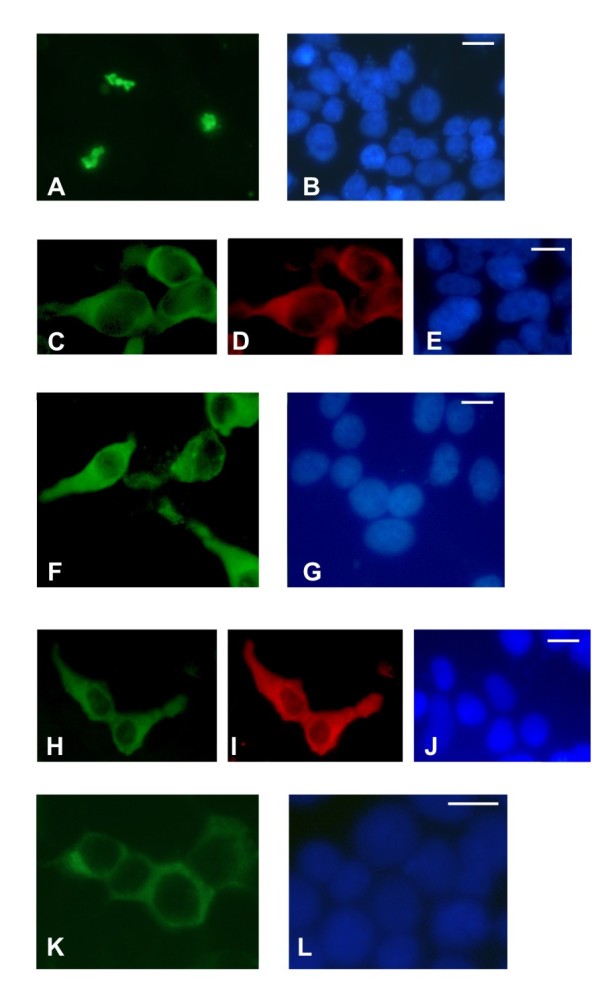
**Intracellular localisation of TSC1 variants detected by immunofluorescence microscopy**. (A - B) Immunofluorescence microscopy of cells expressing the TSC1 E51D variant. (A) Punctate cytoplasmic localisation of the E51D variant; (B) DAPI nuclear stain. White scale bar: 5 μm.(C - E) Immunofluorescence microscopy of cells coexpressing TSC2 and the E51D variant. (C) Diffuse cytoplasmic localisation of the E51D variant in the presence of TSC2; (D) Diffuse cytoplasmic localisation of TSC2; (E) DAPI nuclear stain. White scale bar: 5 μm. (F - G) Immunofluorescence microscopy of cells expressing the TSC1 L50P variant. (F) Diffuse cytoplasmic localisation of the L50P variant; (G) DAPI nuclear stain. White scale bar: 5 μm. (H - J) Immunofluorescence microscopy of cells coexpressing TSC2 and the L50P variant. (H) Diffuse cytoplasmic localisation of the L50P variant in the presence of TSC2; (I) Diffuse cytoplasmic localisation of TSC2; (J) DAPI nuclear stain. White scale bar: 5 μm. (K - L) Immunofluorescence microscopy of cells expressing the TSC1 L50P variant and treated with MG-132. (K) Diffuse cytoplasmic localisation of the L50P variant after MG-132 treatment; (L) DAPI nuclear stain. White scale bar: 5 μm

Exogenous expression of the TSC1 E51D variant resulted in the formation of large, cytoplasmic TSC1 protein aggregates (Figure 4A), consistent with previous results with wild-type TSC1 [31]. Coexpression of TSC2 resulted in a dramatic reduction in the number of aggregates, and both TSC2 and the TSC1 E51D variant were detected throughout the cytoplasm (Figure 4C and 4D). We observed the same expression pattern for the TSC1 R190C, S334L, E478G, Q550E, D658E, A659V and I807T variants (data not shown).

In contrast to wild-type TSC1 and the E51D, R190C, S334L, E478G, Q550E, D658E, A659V and I807T variants, the L50P variant did not form large cytoplasmic aggregates when over-expressed in HEK 293T cells. Instead, the variant was expressed relatively uniformly throughout the cytoplasm (Figure 4F). This localisation was unaffected by coexpression of TSC2 (Figure 4H and 4I) or by MG-132 treatment (Figure 4K). The intracellular localisation patterns of the L61P, L93R, V133F and R190P variants were indistinguishable from the L50P variant (data not shown).

## Discussion

We investigated the effects of 13 putative pathogenic amino acid substitutions on TSC1 function. Our findings are summarised in Table 1. The *TSC1 *c.149T>C (p.L50P), c.182T>C (p.L61P), c.278T>G (p.L93R), c.397G>T (p.V133F) and c.569G>C (p.R190P) changes resulted in reduced levels of TSC1, a reduction in the TSC1-TSC2-dependent inhibition of mTOR activity and a distinct intracellular expression pattern compared to wild-type TSC1. We conclude that these changes are pathogenic. According to the LOVD *TSC1 *mutation database the p.L61P and p.L93R substitutions are probably pathogenic [18]. Our functional analysis supports this conclusion. The p.L50P, p.V133F and p.R190P substitutions are classified as being of unknown pathogenicity in the database. Our data indicates that these changes are pathogenic.

Several studies indicate that *TSC1 *mutations are associated with a less severe clinical presentation in TSC patients [7-9] and further study is required to determine whether patients with a *TSC1 *missense mutation follow this pattern, or have a distinct phenotypic spectrum compared to other TSC patient groups.

The c.153A>C (p.E51D), c.568C>T (p.R190C), c.1001C>T (p.S334L), c.1648C>G (p.Q550E), c.1974C>G (p.D658E), c.1976C>T (p.A659V) and c.2420T>C (p.I807T) substitutions did not affect TSC1 in our assays and were predicted to have no effect on *TSC1 *mRNA splicing. The LOVD *TSC1 *mutation database lists the c.153A>C (p.E51D), c.568C>T (p.R190C) and c.1001C>T (p.S334L) variants as probably not pathogenic, and our functional analysis indicates that they are neutral variants. Furthermore, mutation screening in additional individuals with TSC resulted in the identification of definite pathogenic *TSC2 *mutations in patients carrying the c.153A>C (p.E51D), c.568C>T (p.R190C) and c.1001C>T (p.S334L) substitutions, confirming the classification of these variants as non-pathogenic (data not shown). The LOVD *TSC1 *mutation database lists the c.1648C>G (p.Q550E) and c.2420T>C (p.I807T) variants as being of unknown pathogenicity. Our analysis indicates that these variants are not pathogenic, although we could not confirm experimentally that they do not affect *TSC1 *mRNA splicing.

The c.1433A>G (E478G) substitution is listed as being of unknown pathogenicity and did not affect TSC1 function in our assays. However, one of three splice site prediction programs identified a potential effect of this substitution on splicing. We did not have access to RNA from this particular individual so we compared the activity of the protein product of the predicted splice isoform to wild-type TSC1, the E478G variant and the L117P pathogenic variant. We did not observe any differences between the predicted TSC1 E478GinsGN variant and either wild-type TSC1 or the E478G [see Additional Files 1 and 2]. Therefore, we did not find any evidence that the c.1433A>G substitution was pathogenic.

All of the amino acid substitutions that affected TSC1 function in our assays were in the N-terminal region of TSC1 that was shown by our SIFT analysis to be relatively intolerant of amino acid changes (amino acids 1 - 270; see Figure 1). Two amino acid substitutions from this region, p.E51D and p.R190C, did not affect TSC1 function in our assays. Aspartate (E) and glutamate (D) are acidic amino acids. The BLOSUM 62 and Grantham matrices classify this substitution as conservative (Figure 1A and 1B) and our SIFT analysis predicted that the p.E51D substitution should be tolerated (Table 1). Therefore, in this case, the experimental data were in agreement with the *in silico *predictions. In contrast, the p.R190C substitution was non-conservative according to the BLOSUM 62 and Grantham matrices and was not tolerated according to our SIFT analysis. The predictions were therefore inaccurate for this particular amino acid substitution since the functional analysis did not reveal an effect on TSC1 function, and genetic analysis indicated that the substitution was not pathogenic. This result demonstrates that a conclusion with respect to pathogenicity based on *in silico *analysis only should be made with caution.

To provide clinicians and researchers with the maximum amount of information, gene variant databases should move towards incorporating both *in vitro *and *in silico *data [32]. We will add the functional and *in silico *results on the variants described here to the LOVD *TSC1 *database as part of the individual entries. We will include the BLOSUM 62, Grantham and SIFT scores as well as the potential splice effects of the different variants. However, for all apparent missense variants, conclusions with regard to pathogenicity will continue to rely on clinical, family and functional data. As the above example shows, although *in silico *analysis is a valuable research tool, it is not yet reliable enough to inform clinical decisions.

All the pathogenic TSC1 amino acid substitutions identified to date map between residues 50 and 224 (this study and [11]). One explanation for the apparent clustering of pathogenic amino acid substitutions to this region is that it is critical for TSC1 function. Alternatively, these substitutions may simply be less conservative than the neutral variants we tested. Although the pathogenic substitutions between amino acids 50 and 190 identified in this study were not conservative, there was no clear distinction between these changes and other neutral substitutions. For example, as shown in Figure 1, the neutral p.R190C variant is a non-conservative, non-tolerated substitution that is predicted to have a large effect on TSC1 hydrophobicity, while the pathogenic p.R190P variant is a more conservative substitution and has a less dramatic effect on TSC1 hydrophobicity. Similarly, the p.V133F substitution is more conservative than the p.R190C substitution and has a relatively mild effect on TSC1 hydrophobicity, yet still has a dramatic effect on TSC1 function in our assays. In addition to the p.E51D and p.R190C substitutions, 6 other TSC1 amino acid substitutions did not affect TSC1 function in our assays. The neutral p.S334L and p.E478G substitutions are non-conservative changes located within the substitution-tolerant central region of TSC1 defined by the SIFT analysis (amino acids 270 - 680; see Figure 1). The neutral, non-conservative p.I807T substitution lies outside this central region, but was also predicted by the SIFT algorithm to be tolerated (Table 1). The neutral p.Q550E, p.A659V and p.D658E substitutions are all conservative, tolerated changes. The results of the *in silico *and functional analyses are consistent with the N-terminal of TSC1 being a conserved, substitution-intolerant region that has an important role in maintaining TSC1 expression and activity in the cell.

The L50P, L61P, L93R, V133F and R190P variants were detected at lower levels compared to wild-type TSC1 and the E51D, R190C, S334L, Q550E, D658E, A659V and I807T variants. Inhibition of proteosome-mediated degradation resulted in increased levels of the L50P and L117P variants, indicating that the pathogenic TSC1 variants are normally rapidly degraded by the proteosome. Interestingly, increasing the levels of the TSC1 L50P and L117P variants by inhibiting proteosome activity resulted in an increase in mTOR activity, as estimated by the extent of S6K T389 phosphorylation. Therefore, increased expression of the L50P and L117P variants had a dominant negative effect on mTOR signalling. One possible explanation for this effect is that the mutant variants sequester TSC2 in inactive TSC1-TSC2 complexes, thereby reducing the capacity of the cells to inhibit mTOR activity. Therefore, MG-132 treatment may help distinguish more clearly between pathogenic and non-pathogenic TSC1 variants.

It is unlikely that this dominant negative effect is significant *in vivo *in TSC patients because, as our studies indicate, the L50P and L117P variants are rapidly degraded compared to wild-type TSC1 (Figure 2A). However, it could provide an explanation for the relative scarcity of *TSC1 *missense mutations. It is possible that other missense variants are stable and do have a dominant negative effect on TSC1-TSC2 function *in vivo *that is incompatible with embryonic survival.

Further study is required to explore in more detail how amino acids close to the N-terminal (residues 50 - 224) are important for TSC1 function. One possibility is that they are necessary for the proposed membrane localisation of TSC1 [33]. Alternatively, they may mediate other inter- or intramolecular interactions. Our observation that pathogenic amino acid substitutions prevent the formation of large TSC1-containing aggregate structures suggests that amino acids 50 - 224 may help mediate TSC1 homodimerisation.

## Conclusion

We have analysed 13 *TSC1 *variants identified in TSC patients that cause missense changes to the TSC1 protein, and identified 5 pathogenic substitutions. Our data confirm that functional assays can be used to differentiate between neutral and pathogenic variants, facilitating the identification of pathogenic mutations in individuals with TSC and providing insight into how specific amino acid residues contribute to protein function.

Amino acids close to the N-terminal of TSC1 (amino acids 50 - 190) are essential for TSC1 function. Amino acid changes to this region prevent the formation of large TSC1 aggregates and promote proteosome-mediated degradation of the protein, thereby reducing steady state levels of TSC1 and resulting in increased signalling through mTOR. Rapid degradation of the mutant TSC1 isoforms *in vivo *most likely prevents the possible dominant negative effects of these variants on TSC1-TSC2-dependent inhibition of mTOR signalling.

## Abbreviations

TSC: tuberous sclerosis complex; GAP: GTPase activating protein; mTOR: mammalian target of rapamycin; S6K: p70 S6 kinase.

## Competing interests

The authors declare that they have no competing interests.

## Authors' contributions

MM, MHW and MN performed the practical work; mutation information was provided by DK, JS, AvdO and DH; RE, SP and JdD are responsible for curation of the LOVD *TSC1 *mutation database. The manuscript was drafted by MN and read and approved by all authors.

## Pre-publication history

The pre-publication history for this paper can be accessed here:



## Supplementary Material

Additional file 1 **Additional Figure 1: Inhibition of S6K-T389 phosphorylation by the TSC1 E478GinsGN variant**. Figure showing the inhibition of S6K-T389 phosphorylation by the TSC1. E478GinsGN predicted splice variant Click here for file

Additional file 2 **Figure Legend Additional Figure 1: Inhibition of S6K-T389 phosphorylation by the TSC1 E478GinsGN variant**. Figure legend to Additional Figure 1. Click here for file
