# Supplementary figures and images for: Identification of a region required for TSC1 stability by functional analysis of TSC1 missense mutations found in individuals with tuberous sclerosis complex
Source: BMC Med Genet. 2009 Sep 11;10:88. doi: 10.1186/1471-2350-10-88 (PMC2753308; doi:10.1186/1471-2350-10-88)

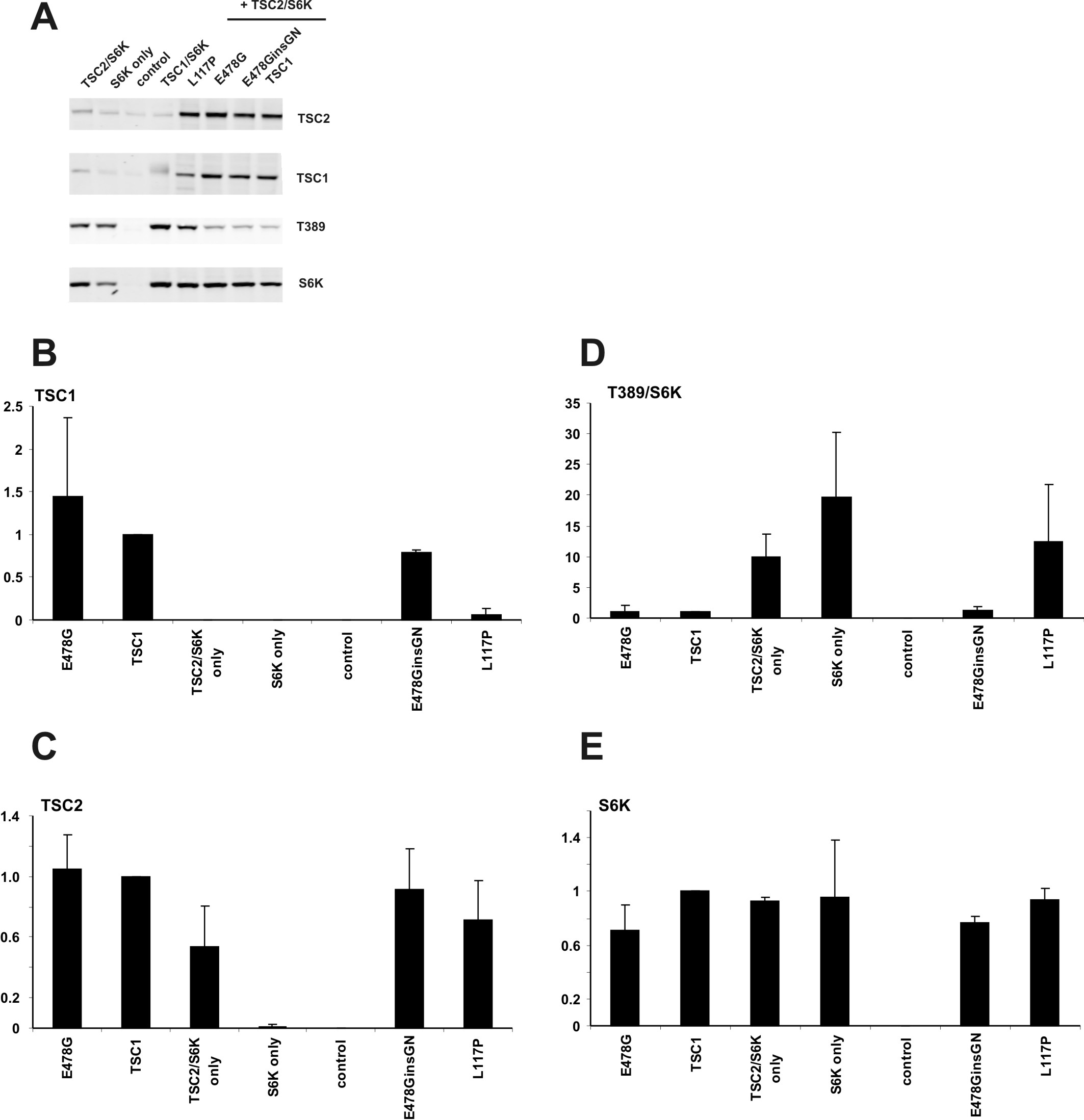

Supplement: Additional file 1 — Additional Figure 1: Inhibition of S6K-T389 phosphorylation by the TSC1 E478GinsGN variant. Figure showing the inhibition of S6K-T389 phosphorylation by the TSC1. E478GinsGN predicted splice variant [file 1471-2350-10-88-S1.jpeg]
