# Supplementary material for: Identification of a region required for TSC1 stability by functional analysis of TSC1 missense mutations found in individuals with tuberous sclerosis complex
Source: BMC Med Genet. 2009 Sep 11;10:88. doi: 10.1186/1471-2350-10-88 (PMC2753308; doi:10.1186/1471-2350-10-88)
Supplement: Additional file 2 — Figure Legend Additional Figure 1: Inhibition of S6K-T389 phosphorylation by the TSC1 E478GinsGN variant. Figure legend to Additional Figure 1. [file 1471-2350-10-88-S2.doc]

**Additional File 2**

**Additional Figure Legend:** Inhibition of S6K-T389 phosphorylation by the TSC1 E478GinsGN variant.

(A) Cells expressing S6K, TSC2 and wild-type TSC1 or the TSC1 L117P, E478G or E478GinsGN variants were analysed by immunoblotting. Levels of TSC1, TSC2, total S6K and T389-phosphorylated S6K were determined using Odyssey^TM^ near infra-red detection (Li-Cor Biosciences) and quantification software. As controls, cells expressing wild-type TSC1 and S6K only (TSC1/S6K), TSC2 and S6K only (TSC2/S6K), S6K only (S6K) or empty vector only (control) were also analysed. S6K and the TSC1 variants were detected with an antibody specific for the myc epitope tag. S6K T389 phosphorylation was reduced in the presence of wild-type TSC1 (TSC1) and the E478G variant compared to the L117P variant. The E478GinsGN variant reduced S6K-T389 phosphorylation as effectively as wild-type TSC1 and the E478G variant. A representative example of 3 separate experiments is shown.

(B) Quantification of the TSC1 signals. The signals for the E478GinsGN, E478G and L117P variants relative to wild-type TSC1 (TSC1) were determined in 3 independent experiments. Standard deviations are indicated.

(C) Quantification of the TSC2 signals. The TSC2 signal in the presence of the E478GinsGN, E478G and L117P variants, relative to the signal in the presence of wild-type TSC1 (TSC1), was determined in 3 independent experiments. Standard deviations are indicated.

(D) Inhibition of S6K T389 phosphorylation in the presence of the E478GinsGN, E478G and L117P variants. The ratio of the S6K T389 phosphorylation signal intensity to the total S6K signal intensity (T389/S6K) was measured in 3 independent experiments and the mean T389/S6K ratios, relative to wild-type TSC1 (wild-type TSC1 T389/S6K ratio = 1) were determined. Standard deviations are indicated.

(E) Quantification of the S6K signals. The total S6K signal in the presence of each of the different TSC1 variants was determined in 3 independent experiments. The signals, relative to the signal in the presence of wild-type TSC1 (TSC1), were determined. Standard deviations are indicated. The total S6K signals indicate that transfection efficiency, gel-loading and blot transfer were relatively constant across all samples.
